# Echinacoside Improves Cognitive Impairment by Inhibiting Aβ Deposition Through the PI3K/AKT/Nrf2/PPARγ Signaling Pathways in APP/PS1 Mice

**DOI:** 10.1007/s12035-022-02885-5

**Published:** 2022-06-04

**Authors:** Hui Qiu, Xuemin Liu

**Affiliations:** Department of Gynaecology and Obstetrics, Shengjing Hospital of China Medical University, Shenyang, 110004 Liaoning China

**Keywords:** Alzheimer’s disease, Echinacoside, Nuclear factor erythroid 2-related factor 2, BACE1, β-Amyloid protein

## Abstract

Echinacoside (ECH), a phenylethanoid glycoside, has protective activity in neurodegenerative disease, including anti-inflammation and antioxidation. However, the effects of ECH in Alzheimer’s disease (AD) are not very clear. This present study investigates the role and mechanism of ECH in the pathological process of AD. APP/PS1 mice treated with ECH in 50 mg/kg/day for 3 months. Morris water maze, nesting test, and immunofluorescence staining used to observe whether ECH could improve AD pathology. Western blot used to study the mechanism of ECH improving AD pathology. The results showed that ECH alleviated the memory impairment of APP/PS1 mice by reducing the time of escape latency as well as increasing the times of crossing the platform and rescued the impaired ability to construct nests. In addition, ECH significantly reduced the deposition of senile plaques in the brain and decreased the expression of BACE1 in APP/PS1 mice through activating PI3K/AKT/Nrf2/PPARγ pathway. Furthermore, ECH decreased ROS formation, GP91 and 8-OHdG expression, upregulated the expression of SOD1 and SOD2 as well as activating the PI3K/AKT/Nrf2 signaling pathway. Moreover, ECH inhibited glia cells activation, pro-inflammatory cytokine IL-1β and TNF-α release, NLRP3 inflammasome formation through TXNIP/Trx-1 signaling pathway. In conclusion, this paper reported that ECH improved cognitive function, inhibited oxidative stress, and inflammatory response in AD. Therefore, we suggest that ECH may considered as a potential drug for AD treatment.

## Introduction

Alzheimer’s disease (AD) is the most common progressive and devastating disease of the elderly that is related to cognition impairment [1]. The pathological features of AD include amyloid beta (Aβ) deposition and intracellular neurofibrillary tangles (NFT) containing hyperphosphorylated tau protein, as well as loss of synapses and neurons [2]. Aβ derived from amyloid precursor protein (APP) by sequential proteolytic cleavages via β-secretase (BACE1) and γ-secretase [3]. BACE1 is a rate-limiting enzyme in the production of Aβ and inhibiting the activity of BACE1 appears to be a prime target for improving AD pathogenesis [4]. Peroxisome proliferator-activated receptor-γ (PPARγ) is a transcription factor that regulates the activity of the BACE1 promoter [4]. A previous study indicated that activating PPARγ could reduce the generation of Aβ and the mechanism related to inhibited BACE1expression [5]. These studies indicate that PPARγ may be involved in production of Aβ.

In addition, oxidative stress and inflammation play essential roles in AD pathogenesis [6, 7]. Oxidative stress participates in AD development by increasing Aβ generation and tau hyperphosphorylation [6], suggesting that antioxidants may be a potential therapy for AD. Nuclear factor erythroid 2-related factor 2 (Nrf2), a transcription factor that regulates the antioxidant and anti-inflammatory response [8], related to AD-mediated cognitive decline [8]. Previous study showed that the expression of Nrf2 was decreased in AD brains [9] and Nrf2 reduction exacerbated cognitive deficits in a mouse model of AD [8]. Activating Nrf2 protects against detrimental stress by promoting the antioxidative defense pathway and ameliorates cognitive impairment in the AD model mouse [10–12]. Therefore, Nrf2 has emerged as a new therapeutic target in AD.

Echinacoside (ECH) is a natural phenylethanoid glycoside that derived from Echinacea angustifolia DC [13]. It performs numerous pharmacological activities, including antioxidant and anti-inflammation, combined with neuroprotective effects [14]. Recent studies have shown that ECH has protective effects on MPTP/MPP^+^-induced neurotoxicity in the mouse model of Parkinson’s disease (PD) by inhibiting inflammatory response and regulating the autophagy pathway [7, 15]. It also plays a protective role in other PD models [16]. Furthermore, ECH extends the lifespan of Caenorhabditis elegans by increasing resistance to oxidative stress and protecting from Aβ-induced toxicity [17]. More importantly, ECH ameliorates the memory impairment and cholinergic deficit induced by Aβ [18]. These studies indicated that ECH had the potency to prevent AD progression. However, the possible therapeutic target of ECH in AD is not clear. Thus, the aims of this study were to testify the effect of ECH on AD pathology in APP/PS1 mice. We demonstrated that long-term treatment with ECH improved the cognitive impairment of APP/PS1 mice by decreasing Aβ production, oxidative stress, and inflammation responses.

## Materials and Methods

### Animals and Treatment

The APPswe/PSEN1dE9 (APP/PS1) transgenic mice, a C57BL6 strain of mice with human APPSwe and PS1-dE9 mutations, were purchased from the Jackson Laboratory and maintained them under standard conditions (room temperature of 22–25 °C and 12 h light/dark cycle). Genotyping was performed by PCR analysis of tail DNA. Five-month-old male mice were randomly divided into two groups (*n* = 6 per group): vehicle and ECH group (50 mg/kg/day). All groups of mice were kept under the same conditions for 3 months before behavior tests were performed. All experimental procedures performed using animals were approved by the Laboratory of Animal Ethical Committee of China Medical University (CMU2020397).

### Behavior Tests

After 3 months of treatment, behavior tests were performed using the Morris water maze and nest building tests. Two days before the test, mice were trained three times per day for two consecutive days with a visible platform. For the next 5 days, a navigation test was carried out. First, the platform was hidden, and both the latency time of mice finding the platform were recorded using the water maze system (ZH0065; Zhenghua Bioequipment, China). In the probe trial, the platform was removed, and the number of times that mice crossed the platform was recorded.

In the nest building test, mice were individually placed in a cage and randomly placed with eight square pieces of paper (5 × 5 cm). The change in the pieces of paper was observed for 7 days and photographically recorded. The nest-building abilities were assessed according to the scoring criteria: 1 = no biting/tearing, with random dispersion of the paper; 2 = no biting/tearing of paper, with gathering in a corner/side of the cage; 3 = moderate biting/tearing of paper, with gathering in a corner/side of the cage; and 4 = extensive biting/tearing of paper, with gathering in a corner/side of the cage.

### Tissue Preparation

At the end of the behavior tests, the mice were anesthetized and perfused with PBS. After that, the brains were quickly collected on ice, and one hemisphere was frozen and stored at − 80 °C, and the other was immersion-fixed in 4% paraformaldehyde for the histological study.

### Measurement of SOD Activity and ROS

The cerebral cortex was homogenized and centrifuged at 4 °C. The supernatant was collected to assay for protein concentration by BCA protein assay kit. After the protein concentration was measured, the activity of superoxide dismutase (SOD) was determined by SOD assay kit according to the manufacturer’s instructions (Najing Jiancheng A001-3–2). Cell suspension of fresh tissues was incubated with DCFH-DA (Najing Jiancheng E004-1–1) for 30 min at 37 ℃, and the fluorescence intensity of reactive oxygen species (ROS) was detected by enzyme labeling instrument.

### Immunofluorescence Staining

The frozen sections were blocked with 5% goat serum for 30 min and incubated with anti-Aβ antibody (mouse monoclonal; 1:100, Santa Cruz) or anti-Aβ antibody/anti-GFAP antibody (rabbit polyclonal; 1:200, Cell signal)/anti-Iba-1 (rabbit polyclonal; 1:200, Wako) overnight at 4 °C. The sections were washed with PBS and then incubated with Alexa Fluor 488- or Alex Fluor 594-conjugated secondary antibodies for 2 h. Images were acquired using the laser scanning confocal microscope (TCS SP8, Leica, Germany).

### Quantitative RT-PCR

Total RNA of mouse tissues was extracted using Total RNA Kit according to the manufacturer’s instruction and reverse transcribed into cDNA. The cDNA synthesis conditions were 37 °C for 15 min and then 85 °C for 5 s. Quantitative real-time PCR was performed in the 7300 Sequence Detection System using the SYBR Green PCR Master mix (AG11702, Accurate Biology, China). At least three independent assays of each cDNA sample were conducted. The primers and probes used in PCR are listed as follows:

TNF-α:

F-AGCCCCCAGTCTGTATCCTT

R-ACAGTCCAGGTCACTGTCCC

IL-1β:

F-AGCCAAGCTTCCTTGTGCAAGTGT

R-GCTCTCATCAGGACAGCCCAGGT

BACE1:

F-GGAACCCATCTCGGCATCC

R-TCCGATTCCTCGTCGGTCTC

SOD1:

F-AACCAGTTGTGTTGTCAGGAC

R-CCACCATGTTTCTTAGAGTGAGG

GAPDH:

F-TGCAGTGGCAAAGTGGAGAT

R-TTTGCCGTGAGTGGAGTCATA

The gene expression values were normalized to those of GAPDH.

### Western Blot

Mouse tissues were lysed in RIPA lysis buffer on ice for 3 h and then were centrifuged at 12,000* g* for 15 min at 4 °C to collect the supernatant. The protein concentrations were measured using the Bradford assay kit. Protein, 30 μg per lane, was separated by 10% SDS-PAGE gel and then transferred to PVDF membranes. Membranes were blocked with 5% nonfat milk for 30 min and incubated with primary antibodies for oligomer (rabbit polyclonal; 1:1000, Sigma), ADAM10 (rabbit polyclonal; 1:1000, Abcam), BACE1 (rabbit polyclonal; 1:1000, Abcam), PS1 (rabbit polyclonal; 1:1000, Cell Signal), PEN2 (rabbit polyclonal; 1:1000, Cell Signal), Nicastrin (NCT) (rabbit polyclonal; 1:1000, Cell Signal), APH-1 (rabbit polyclonal; 1:1000, Thermo), Nrf2 (rabbit polyclonal; 1:1000, Abcam), HO-1 (rabbit polyclonal; 1:1000, Abcam), GP91 (rabbit polyclonal; 1:1000, Abcam), SOD1 (rabbit polyclonal; 1:1000, Proteintech), SOD2 (rabbit polyclonal; 1:1000, Proteintech), TXNIP (rabbit polyclonal; 1:1000, Abcam), NLRP3 (rabbit polyclonal; 1:1000, Abcam), Trx-1 (rabbit polyclonal; 1:1000, Abcam), p-AKT (rabbit polyclonal; 1:1000, Cell Signal), AKT (rabbit polyclonal; 1:1000, Cell signal), p-PI3K (rabbit polyclonal; 1:1000, Cell Signal), PI3K (rabbit polyclonal; 1:1000, Cell Signal), GAPDH (rabbit polyclonal; 1:10,000, Cell Signal), p-PPARγ (rabbit polyclonal; 1:1000, Cell Signal), and PPARγ (rabbit polyclonal; 1:1000, Cell Signal) overnight at 4 °C. After washing three times for 15 min, the membranes were incubated with HRP-conjugated secondary antibody for 1.5 h and detected using ECL.

### Statistical Analysis

All data are represented as mean ± standard error of the mean (SEM). Statistical significances between the ECH treatment group and the vehicle control treatment group were determined by *t*-test. The difference was considered to be statistically significant when *P* < 0.05.

## Results

### ECH Improved Memory Deficits in APP/PS1 Mice

**Fig. 1:**
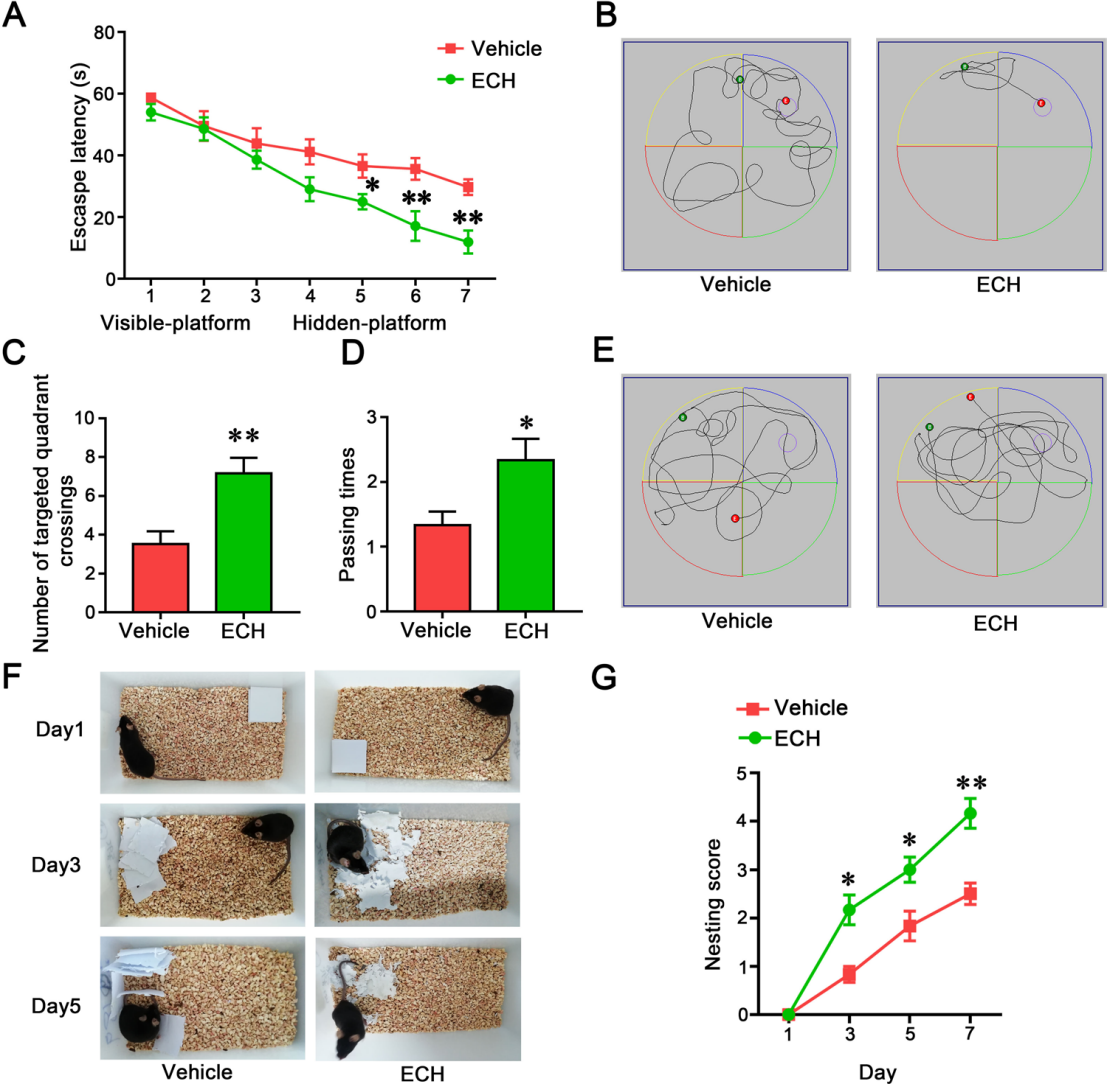
ECH treatment improved the cognitive capacity of APP/PS1 mice. APP/PS1 mice (5 months old) were treated with ECH (i.p., 50 mg/kg/day) for 3 months. Morris water maze tests with 2 days of visible platform training, 5 days of hidden platform testing, and a probe trial after 24 h of the last hidden platform test were used to evaluate cognitive ability. **A**, **B** Mice from different groups exhibited a similar escape latency to the visible platform on days 1–2, and the hidden platform tests showed that ECH administration decreased the escape latency from the 3rd to 7th day. **C** A representative path showing the mouse performance in the hidden platform trail on the 5th day. **D**, **E** In the probe trial, ECH treatment significantly increased the times of crossing the platform’s former location. Nest construction was visualized after Morris water maze tests. **F**, **G** ECH treatment significantly rescued the impaired ability to construct nests. Data were presented as the mean ± SEM; *n* = 6, *P < 0.05; **P < 0.01

To address whether ECH could improve memory impairments in APP/PS1 mice, we tested the mice using the Morris water maze and nest building tests (Fig. 1), which assessed spatial learning and memory function. The results showed that the latency time of mice finding the platform were significantly reduced in ECH treatment groups on the navigation test. The platform crossing times were significantly higher in the probe trial compared with the vehicle group (Fig. 1A–E). Moreover, ECH could improve the ability of nest construction compared with the vehicle group (Fig. 1F, G). The results suggest that ECH can significantly improve learning and memory dysfunction in APP/PS1 mice.

### ECH Inhibited the Production of Aβ_1-42_ Production as Detected by Immunofluorescence Staining

**Fig. 2:**
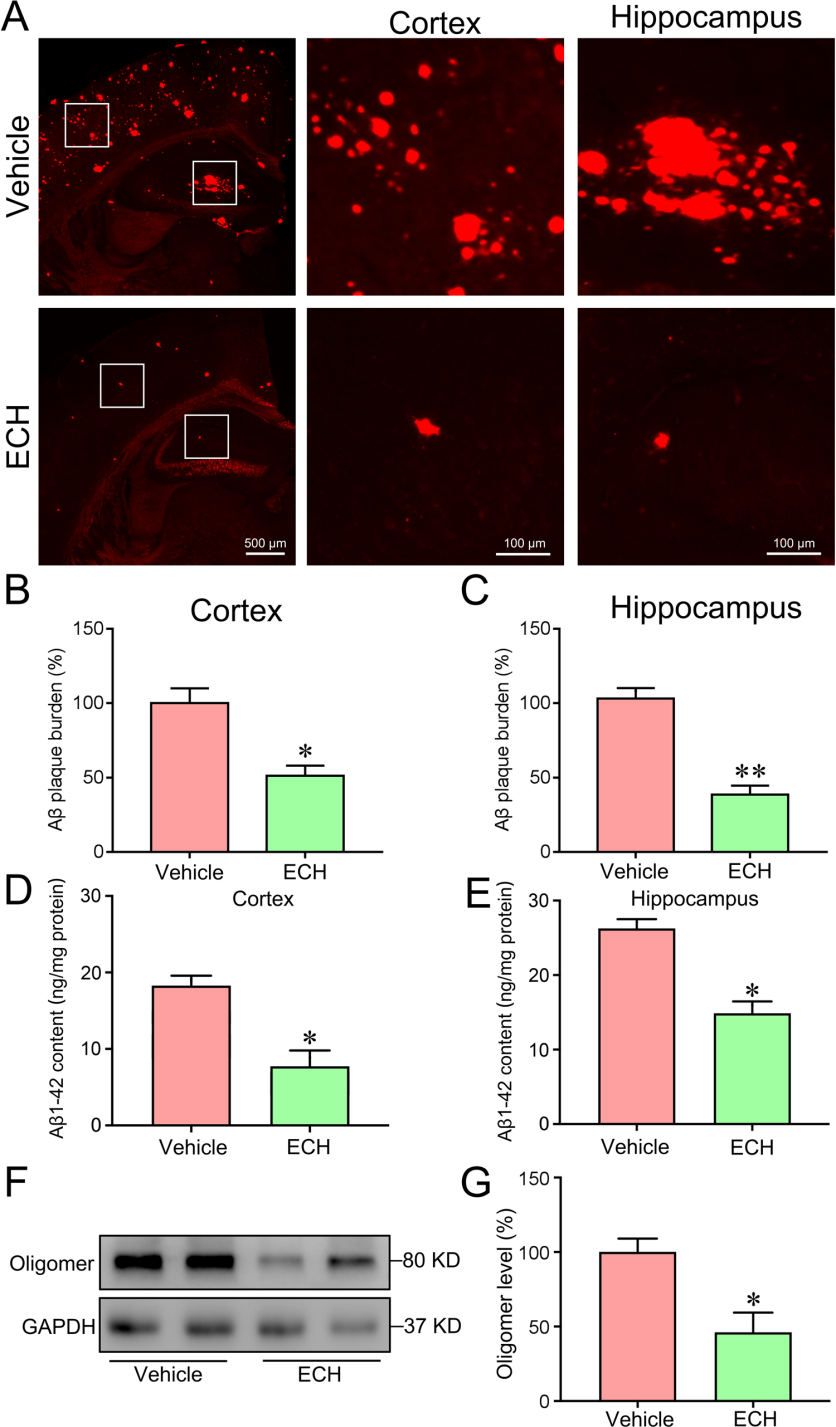
ECH treatment reduced senile plaque burden in APP/PS1 mice. Five-month-old APP/PS1 mice were treated with ECH for 3 months. **A** Immunofluorescent labeling of Aβ showing the Aβ plaque in the cortex and hippocampus of APP/PS1 mice. **B**, **C** Quantification of Aβ fluorescence revealed a reduced number of Aβ plaques in the cortex and hippocampus of APP/PS1 mice after ECH treatment. **D**, **E** ELISA showed that content of Aβ1-42 in the cortex and hippocampus. **F**, **G** Immunoblot analysis reveals the expression level of Aβ oligomer. Data were presented as the mean ± SEM; *n* = 6, *P < 0.05; **P < 0.01

To determine the mechanism of ECH to improve the learning and memory abilities of mice, senile plaque deposition was detected by immunofluorescence staining. The results showed that the number of Aβ_1-42_ in the cortex and hippocampus of mice decreased significantly compared with the vehicle group (Fig. 2), indicating that ECH alleviated cognitive impairment by reducing the production of Aβ_1-42_.

### ECH Reduced BACE1 Expression in the APP/PS1 Mice

**Fig. 3:**
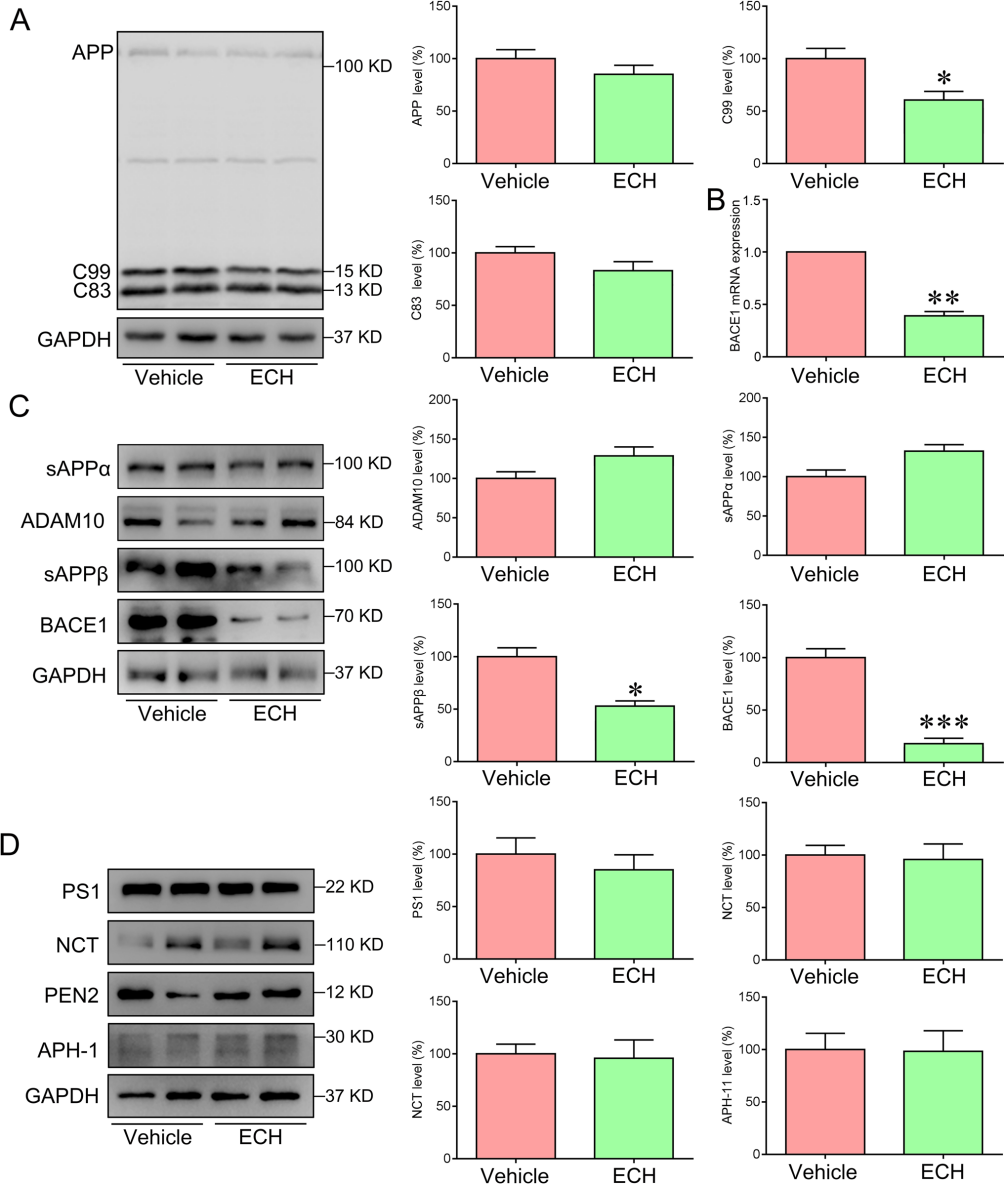
ECH inhibited the activation of BACE1 and the secretion of sAPPβ in the cortex of APP/PS1 mice. Five-month-old APP/PS1 mice were treated with ECH for 3 months. **A** Immunoblot analysis showed the expression levels of APP, C83, and C99 in the cortex. **B** The mRNA expression level of BACE1 in the cortex. **C** Immunoblot analysis showed the expression levels of ADAM10, BACE1, the secretion of sAPPα and sAPPβ in the cortex. **D** Immunoblot analysis reveals the expression level of the subunits of γ-secretase, including PS1, NCT, PEN2, and APH-1. Data were presented as the mean ± SEM; *n* = 6, *P < 0.05; **P < 0.01; ***P < 0.001

Aβ is produced by the APP through the amyloid cleavage pathway, while the APP non-amyloid pathway can inhibit the production of Aβ. Therefore, the amyloid pathway cleavage enzyme BACE1 and non-amyloid cleavage enzyme ADAM10, as well as their production of cleavage were assessed. The results showed that the full of APP has no change compared with vehicle (Fig. 3A). However, there was a significant decrease in the expression of BACE1 and production of cleavage sAPPβ and C99 after ECH treatment (Fig. 3A–C). However, the expression of ADAM10, sAPPα and γ-secretase (PS1, NCT, PEN2, and APH-1) were not significantly different compared with the vehicle group (Fig. 3B–D). These results suggested that ECH administration could down-regulate the level of BACE1 to inhibit Aβ generation.

### ECH Significantly Inhibited Oxidative Stress

**Fig. 4:**
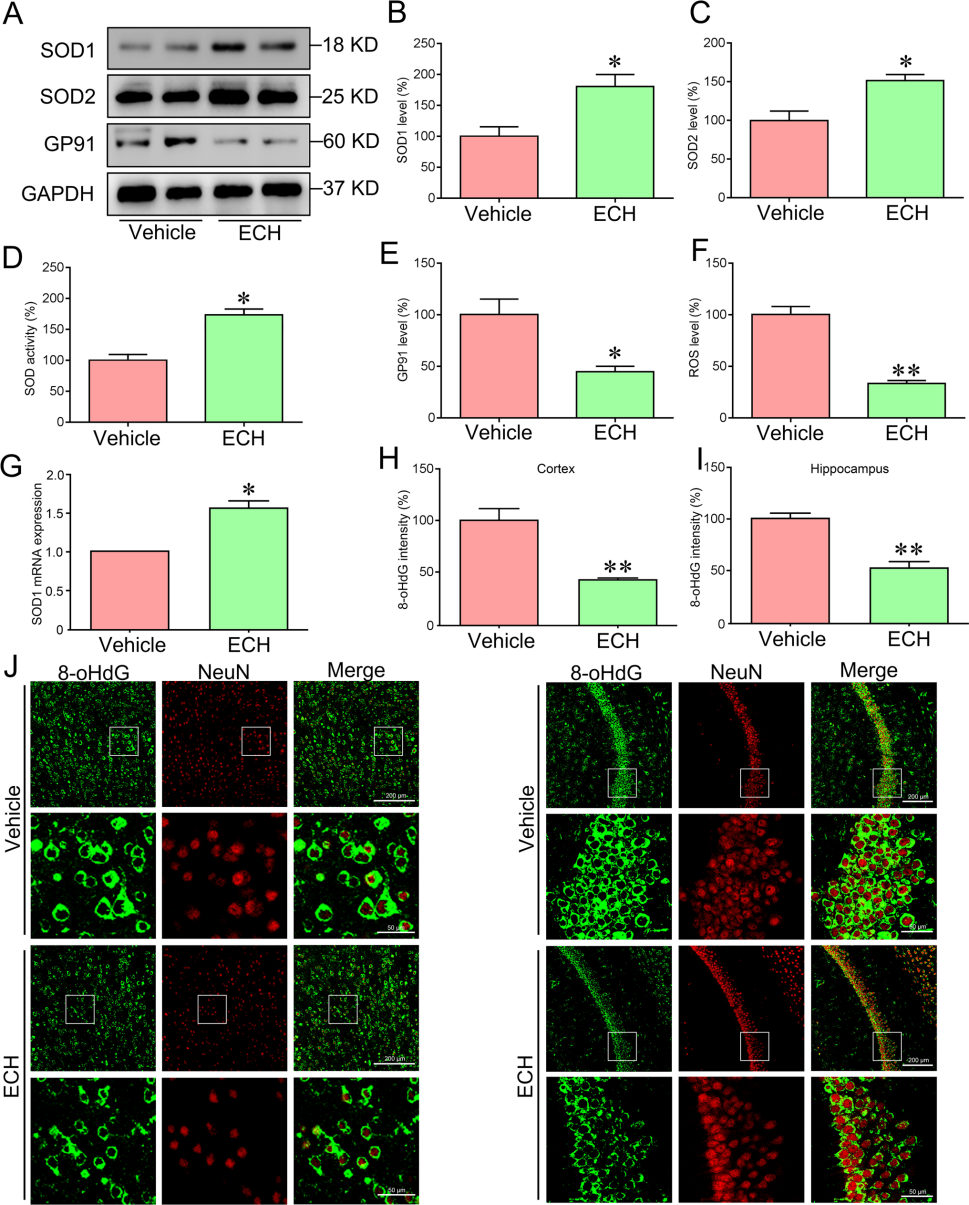
ECH treatment limited the oxidative stress in the cortex of APP/PS1 mice. Five-month-old APP/PS1 mice were treated with ECH for 3 months. **A**–**C**, **E** Immunoblot analysis showed the expression level of SOD1, SOD2, and GP91 in the cortex. **D** Changes in SOD activity in the cortex. **F** ROS production in the cortex was detected with the dichlorofluorescein diacetate probe. **G** The mRNA expression level of SOD1 in the cortex. **H**–**J** Immunofluorescent labeling of 8-oHdG showing the 8-oHdG in the cortex and hippocampus of APP/PS1 mice. Data were presented as the mean ± SEM; *n* = 6, *P < 0.05; **P < 0.01

Oxidative stress plays a vital role in AD pathogenesis and promotes AD development via increasing Aβ deposition and tau hyperphosphorylation [7]. Therefore, antioxidant drugs may have a therapeutic effect on AD. We measured ROS and antioxidant enzyme activity to investigate the relationship between ECH and oxidative stress. After ECH treatment, the content of ROS significantly decreased, while antioxidant enzyme (SOD1 and SOD2) activity increased significantly (Fig. 4A–D, F, G).

Oxidative stress-induced damage occurs to the lipids of cellular membranes, proteins, and DNA [19]. 8-Hydroxy-2′-deoxyguanosine (8-OHdG), one of the primary forms of free radical-induced oxidative damage, has been recognized as a biomarker of oxidative stress [19]. Moreover, GP91 has also been recognized as biomarkers of oxidative stress [20]. In this study, the levels of GP91 and 8-OHdG were significantly decreased after ECH treatment (Fig. 4A, E, H–J). Taken together, these results illustrated that ECH could significantly reduce oxidative stress, and its mechanism might be related to increase the activity of antioxidant enzymes.

### ECH Restrained Glia Cell Activation and Pro-inflammatory Cytokine Release

ECH alleviates LPS-induced cell apoptosis and inflammation in rat intestinal epithelial cells [21] and possesses a neuroprotective effect via inhibiting inflammation. However, it is unclear how ECH improves AD pathology by anti-inflammation in the APP/PS1 mice.

**Fig. 5:**
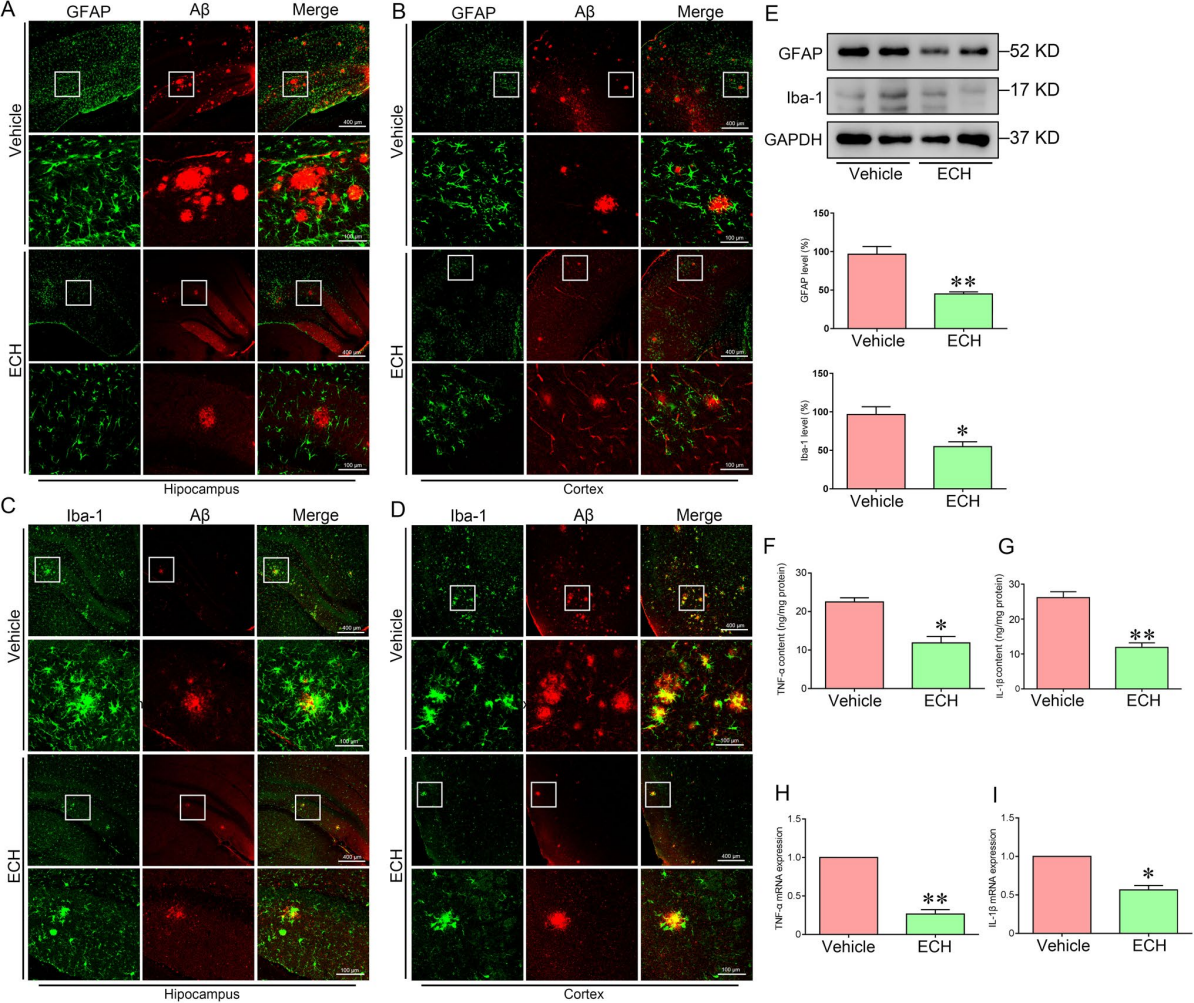
ECH suppressed the neuroinflammatory reaction in the cortex and hippocampus of APP/PS1 mice. Five-month-old APP/PS1 mice were treated with ECH for 3 months. **A**–**D** ECH treatment suppressed the activation of microglia and astrocytes around the Aβ plaque. **E** Immunoblot analysis showed the expression levels of GFAP and Iba-1. **F**, **G** ELISA showed that content of IL-1β and TNF-α in the cortex. **H**, **I** The mRNA expression of IL-1β and TNF-α. Data were presented as the mean ± SEM; *n* = 6, *P < 0.05; **P < 0.01

GFAP or Iba-1 and Aβ were stained with double-labeled immunofluorescence, and we found that ECH could significantly inhibit the activation of glial cells around senile plaques (Fig. 5A–D). Western blotting results showed that ECH inhibited the expression of GFAP and Iba-1 compared with the vehicle group (Fig. 5E).

To examine whether ECH inhibits inflammatory responses in APP/PS1 mice, pro-inflammatory cytokines such as TNF-α and IL-1β were detected by ELISA and real-time polymerase chain reaction (RT-PCR) (Fig. 5F–I). The results suggest that ECH inhibited the expression of TNF-α and IL-1β to decrease the inflammation response.

### ECH Activated PI3K/AKT/Nrf2/PPARγ Signaling Pathways

**Fig. 6:**
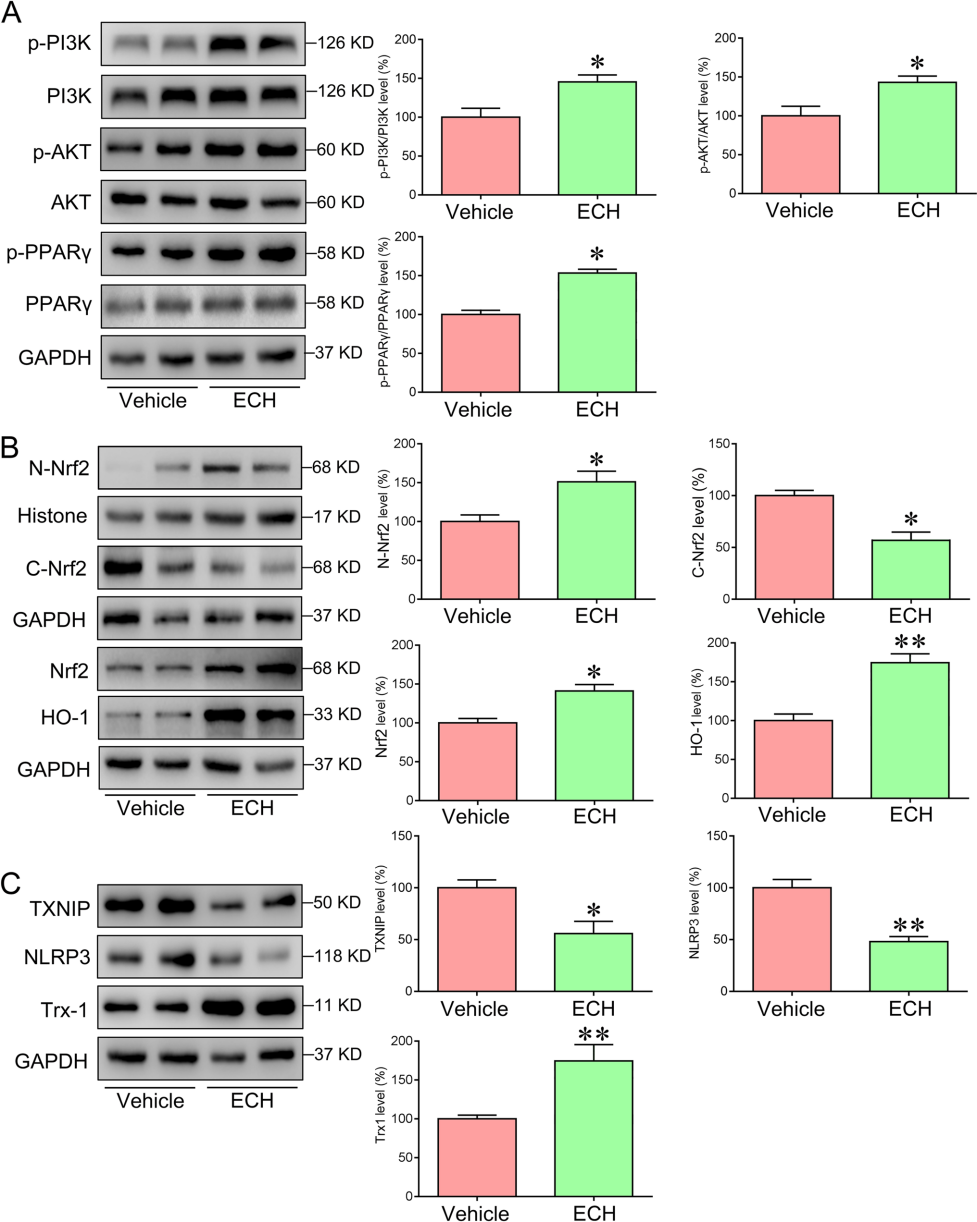
ECH activated the PI3K/AKT/Nrf2/PPARγ signaling pathways in the cortex of APP/PS1 mice. Five-month-old APP/PS1 mice were treated with ECH for 3 months. **A** Immunoblot analysis showed the expression levels of p-PI3K, PI3K, p-AKT, AKT, p-PPARγ, and PPARγ in the cortex. **B** Immunoblot analysis showed the expression level of Nucleu-Nrf2, Nrf2, and HO-1 in the cortex. **C** Western blot analysis showed the expression level of TXNIP, NLRP3, and Trx-1 in the cortex. Data were presented as the mean ± SEM; *n* = 6, *P < 0.05; **P < 0.01

Transcription factor PPARγ regulates the activity of the BACE1 promoter, and activating PPARγ can inhibit BACE1 [5, 16]. As an upstream signal molecule of PPARγ, deficiency of Nrf2 attenuated PPARγ transcriptional activity [22]. Previous study indicated that PI3K/AKT signaling pathway was participated in activating Nrf2 [23]. In our study, PI3K, AKT, Nrf2, PPARγ, and HO-1 expression were assessed. The results showed that ECH increase the phosphorylation the levels of PI3K and AKT, promoted Nrf2 expression in the nucleus and cytoplasm, subsequently promote PPARγ expression compared with the vehicle group (Fig. 6A, B). Moreover, ECH accelerated the expression of HO-1 (Fig. 6B). In conclusion, our results indicate that ECH improves AD pathology by activating PI3K/AKT/Nrf2/PPAPγ pathways.

### ECH Inhibited NLRP3 Inflammasome Activation Through TXNIP/Trx-1 Signaling Pathway

Moreover, Nrf2 is related to inflammation by inhibiting NLRP3 inflammasome activation through TXNIP/Trx-1 complex regulation [11]. In our research, we found that the expression of NLRP3 inflammasome significantly decreased and Trx-1 expression increased after ECH treatment. However, the lever of TXNIP was down-regulated by ECH (Fig. 6C). Our results showed that ECH inhibited NLRP3 inflammasome activation by regulating TXNIP/Trx-1 pathway.

## Discussion

In this study, we investigated the molecular mechanisms of ECH on AD using APP/PS1 mice. We found that ECH improves mouse cognitive function and reduces both senile plaque deposition and oxidative damage in the brain.

Our data demonstrated that ECH ameliorated memory impairments and decreased Aβ generation in APP/PS1 mice. We found that ECH reduced Aβ production by inhibiting the expression of BACE1, but there were no effects on α- or γ-secretase. This finding is consistent with the research of Dai et al. [24].

BACE1 expression is regulated by a variety of transcription factors, including NF-κB and PPARγ [4]. It has been reported that the level of PPARγ significantly reduced in the brains of AD patients and mice models [25]. PPARγ depletion enhances BACE1 mRNA levels by promoting BACE1 gene promoter activity. Conversely, overexpression of PPARγ or PPARγ activators, reduced BACE1 gene promoter activity and inhibited BACE1 expression [26], which ultimately reduces the generation of Aβ [5]. Although our results showed that ECH significantly inhibited the expression of BACE1, it was unclear whether the mechanism of ECH is involving in activating PPARγ. In our study, we found that ECH can activate PPARγ, leading to the down-regulation of BACE1 and the inhibition of Aβ. Furthermore, PPARγ as a direct downstream transcriptional target of Nrf2, its promoter activity can be activated by Nrf2. On the contrary, Nrf2 deficiency also leads to decreased expression of PPARγ [22]. A decline in the expression of the transcription factor Nrf2 have been observed in AD brains and Nrf2 induction ameliorates cognitive impairment in the AD model mouse [12, 27]. To assess whether ECH activate PPARγ by promoting Nrf2 expression, thereby protecting AD model mice against disease progression, we detected Nrf2 expression in the nucleus and cytoplasm. The results showed that ECH promoted Nrf2 translocation to the nucleus and the expression of downstream genes PPARγ. A previous study showed that PI3K/AKT pathway is related to Nrf2 activation [23]. After treatment with the PI3K/AKT inhibitor Ly294002, the p-AKT and Nrf2 levels, as well as the Nrf2 nuclear translocation, were significantly suppressed [23]. Anthocyanins (potent antioxidant and neuroprotective agents) reduce Aβ oligomer-induced neurotoxicity by the PI3K/AKT/Nrf2 pathway [28]. Panax notoginseng saponins activated Nrf2 in a PI3K/AKT pathway-dependent manner and protected against barrier disruption [29]. In this study, we determined whether ECH could increase Nrf2 nuclear translocation by activating the PI3K/AKT pathway. Our results show that ECH up regulates the phosphorylation level of PI3K and then induces AKT to activate Nrf2. The activated Nrf2 can increase the promoter activity of PPARγ and inhibit the expression of BACE1.

Accumulating evidence indicates that AD is related to oxidative stress [30]. Our study found that the antioxidant enzyme activity of SOD1 and SOD2 increased after ECH treatment, and SOD1 expression was upregulated. The levels of the oxidative stress biomarkers (GP91 and 8-OHdG) were significantly decreased after ECH treatment. Furthermore, ECH inhibited glia cells activities and then reduced the release of inflammatory cytokines, including IL-1β and TNF-α. Nrf2 acted as the key transcriptional regulator [31], against oxidative stress damage in AD [25] by increasing the endogenous antioxidant capacity [32]. In addition, cytoprotective gene heme oxygenase 1 (HO-1) could be induced by Nrf2 nuclear translocation. The Nrf2/HO-1 signaling axis is a multiple organ protection chain that protects against oxidative stress injury. Nrf2/HO-1 axis can increase SOD2 expression and modulate mitochondrial structure and function in order to resist oxidative stress injury. In this study, we found that ECH can promote Nrf2 translated into the nucleus, then induce the expression of HO-1, up regulate the expression of antioxidant enzyme SOD2 and inhibit oxidative stress. Recently, studies have shown that Nrf2 induction increased GSH levels and attenuated reactive astrocytosis [33]. Resveratrol attenuates oxidative stress by PI3K/AKT-induced Nrf2 activation [34]. Panax notoginseng saponins activated antioxidant signaling by Nrf2 in a PI3K/AKT pathway-dependent manner and protected against barrier disruption [29]. Our results indicated that ECH inhibited oxidative stress damage by PI3K/AKT-induced Nrf2 activation pathway.

In addition, chronic inflammation has a vital role in the onset and progression of AD [35]. Nrf2, thioredoxin interacting protein (TXNIP) and nucleotide-binding oligomerization domain-like receptor protein 3 (NLRP3) inflammasome pathways are closely related to inflammation-related diseases [36]. The NLRP3 inflammasome is a complex of multi-proteins that regulate inflammation by activating the secretion of the pro-inflammatory cytokine [37]. TXNIP as an endogenous regulator can interact with NLRP3, which activates the NLRP3 inflammasome and promotes inflammatory responses [38]. A previous study showed that Nrf2 inhibits NLRP3 inflammasome activation by regulating the TXNIP/Trx-1 complex [39]. DI-3-n-butylphthalide treatment suppresses TXNIP-NLRP3 interaction and inhibits NLRP3 inflammasome activation by upregulating Nrf2 [11]. Our results suggested that ECH decreased the TXNIP level and inhibited NLRP3 inflammasome activation against the neuroinflammation associated with Nrf2.

## Conclusion

**Fig. 7:**
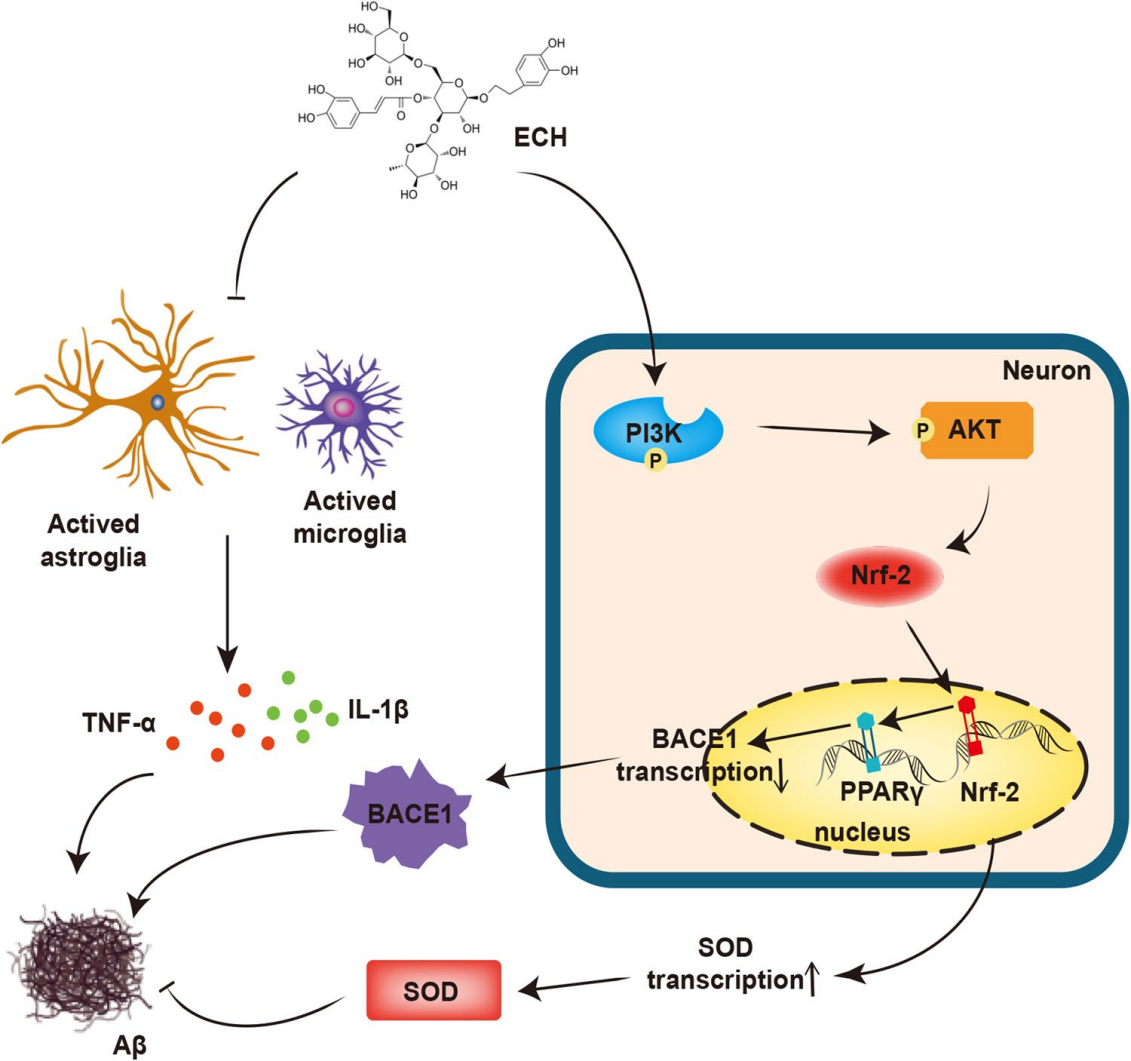
Schematic diagram shows the mechanism of ECH ameliorated AD pathological process. ECH inhibits BACE1 expression and oxidative stress by activating PI3K/AKT/Nrf2/PPARγ signaling pathways, thus reducing Aβ production. In addition, ECH can reduce the production of Aβ by inhibiting the release of inflammatory factors

ECH decreased the Aβ deposition by reducing the expression of BACE1 through activating PI3K/AKT/Nrf2/PPARγ signaling pathway. Furthermore, ECH promoted Nrf2 via the PI3K/AKT signaling pathway against oxidative stress, inhibited TXNIP-NLRP3 interaction, reduced NLRP3 inflammasome against inflammation, and improved AD pathology (Fig. 7).

## Data Availability

The datasets used and/or analyzed during the current study are available from the corresponding author on reasonable request.
